# Ultra-small curcumin-ruthenium coordination polymer nanodots prevent renal ischemia-reperfusion injury and the progression to chronic kidney disease

**DOI:** 10.3389/fbioe.2024.1506909

**Published:** 2025-01-14

**Authors:** Xian Liu, Qin Yu, Hai-Bo Mao, Jing-Bo Hu, Wei-Hua Liu

**Affiliations:** ^1^ Department of Urology, Beilun People’s Hospital, Ningbo, Zhejiang, China; ^2^ Faculty of Materials Science and Chemical Engineering, Ningbo University, Ningbo, China

**Keywords:** acute kidney injury, chronic kidney disease, ischemia-reperfusion, curcumin, ruthenium

## Abstract

Renal ischemia-reperfusion (IR) induces tissue hypoxia, resulting in disrupted energy metabolism and heightened oxidative stress. These factors contribute to tubular cell damage, which is a leading cause of acute kidney injury (AKI) and can progress to chronic kidney disease (CKD). The excessive generation of reactive oxygen species (ROS) plays a crucial role in the pathogenesis of AKI. This study presents the synthesis of curcumin ultra-small coordination polymer (Ru/Cur) nanodots and their application in scavenging ROS in renal tissues. By adding ruthenium ions to a methanol solution containing the natural product curcumin, ultra-small Ru/Cur nanodots were successfully synthesized. To enhance the dispersibility of these nanoparticles in water, polyvinylpyrrolidone (PVP) was used as a growth aid, resulting in highly stable nanodots with sizes smaller than 10 nm. The results indicated that Ru/Cur nanodots effectively eliminated various ROS and demonstrated significant therapeutic effects and biocompatibility in IR-AKI mice, reducing markers of kidney function damage, alleviating renal oxidative stress, and decreasing inflammatory cell infiltration. Ru/Cur nanodots inhibited renal fibrosis by suppressing epithelial-mesenchymal transition and the secretion of transforming growth factor-β1 in the model of IR-AKI to chronic kidney disease (CKD). In summary, our findings confirm that Ru/Cur nanodots mitigate the pathological conditions associated with both AKI and its progression to CKD by reducing IR-induced tubular cell injury.

## 1 Introduction

Acute kidney injury (AKI), also known as acute renal failure, is defined as the sudden loss of renal function characterized by a rapid increase in serum creatinine (CRE) and blood urea nitrogen (BUN) levels (Li and Xing, 2024). As one of the most vital metabolic and excretory organs, the kidneys are susceptible to a variety of insults, with ischemia-reperfusion (IR)-induced hypoxia and nephrotoxic drugs being among the most common culprits (O'Neal et al., 2016). Presently, there are no definitively reliable treatment modalities to address AKI or prevent its progression to chronic kidney disease (CKD) (Maeda et al., 2024). Clinical management primarily revolves around supportive care and renal replacement therapy, resulting in approximately 5% of AKI patients experiencing persistent renal dysfunction necessitating long-term dialysis or leading to mortality.

In the process of IR-AKI, the electron transport chain of mitochondria is prone to interference by various factors, leading to electron leakage and ROS production. Therefore, mitochondria are considered the primary source of ROS. Excessive ROS can disrupt biological membranes, resulting in dysfunction such as membrane rupture, mitochondrial swelling, and dissolution. Antioxidant therapy strategies can effectively alleviate mitochondrial damage, reduce excessive ROS levels within mitochondria, ultimately delaying or even reversing the progression of AKI (Chen et al., 2023). Currently, there are many types of antioxidants used in clinical practice, such as N-acetylcysteine, vitamins, amino acids and their derivatives, omega-3 polyunsaturated fatty acids, curcumin, and so on (Wang et al., 2021; Xu et al., 2018). The effectiveness of treatments for AKI is often unsatisfactory because the drugs used can be limited by the glomerular filtration system, preventing them from reaching the damaged tubules effectively. To improve antioxidant therapy for AKI, it is important to maximize the distribution of antioxidant drugs in renal tissues, ensure effective distribution in renal tubular epithelial cells, and increase drug concentrations for treatment.

Curcumin (Cur), a small molecule polyphenolic compound, possesses numerous important biological functions, such as anti-inflammatory, antioxidant, and immunomodulatory pharmacological actions (Cai et al., 2022). It was reported that Cur could effectively protect renal tubular epithelial cells from oxidative stress induced by H_2_O_2_, playing a crucial role in antioxidant defense. However, due to factors such as poor water solubility, low *in vivo* levels, and low bioavailability, its clinical application is limited, with minimal therapeutic efficacy. Modifying Cur through formulation methods to improve its *in vivo* distribution, increase solubility, and enhance bioavailability can lead to targeted antioxidant therapy for AKI.

**FIGURE 1:**
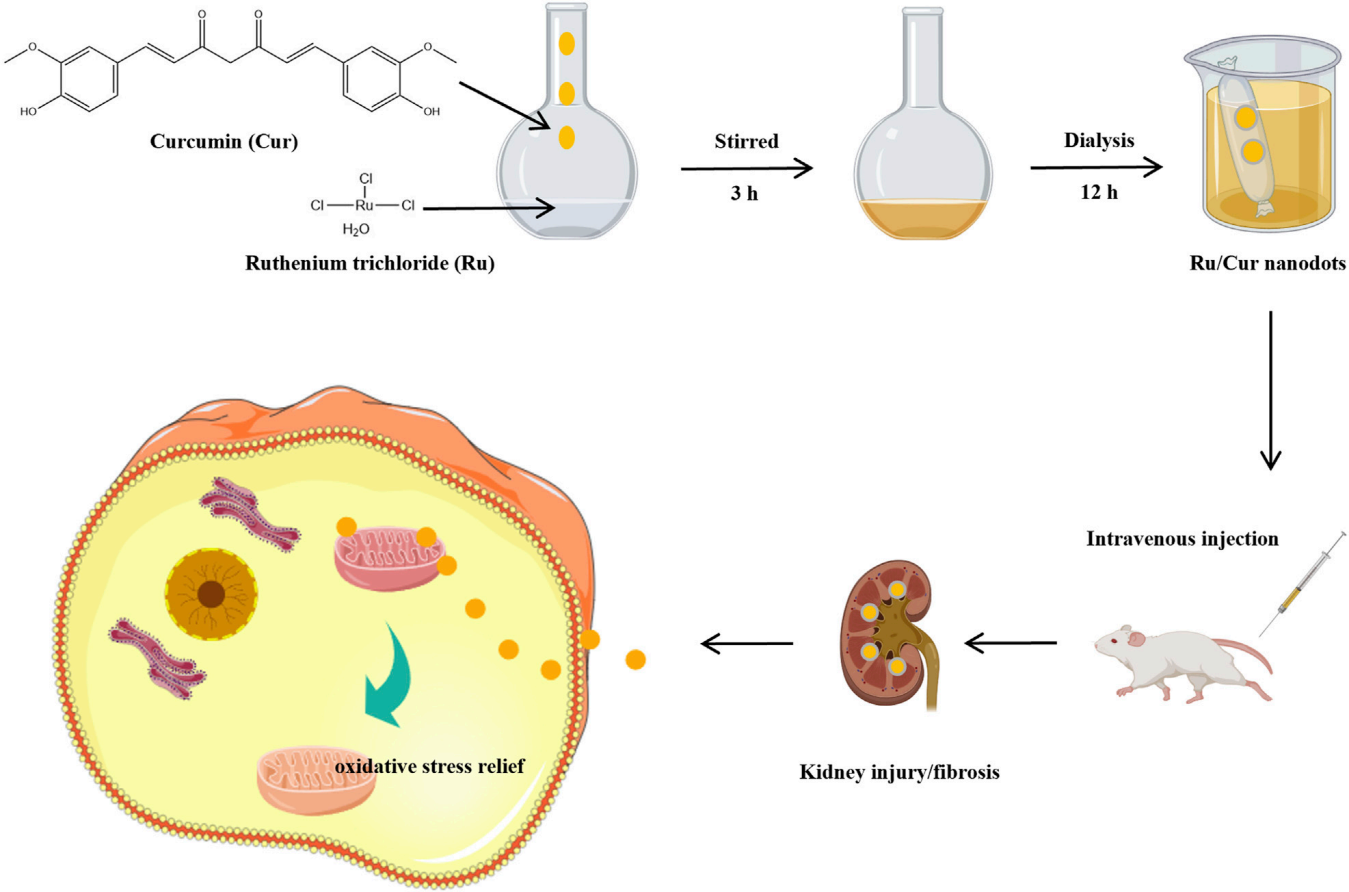
Schematic diagram of the synthesis of ultra-small ruthenium/curcumin nanodots and its use in ischemia-reperfusion-induced acute kidney injury (IR-AKI) and the progression from AKI to chronic kidney disease (CKD).

In this study, we utilized Cur and a multivalent rare metal, ruthenium (Ru), to synthesize ultra-small Ru/Cur nanodots through metal-organic coordination, enabling them to circumvent the constraints of the glomerular filtration system and effectively reach the renal tubules (Figure 1). The physicochemical properties of Ru/Cur nanodots were characterized, and the mechanism protecting proximal tubule cells from damage was also investigated. Additionally, the therapeutic potential of Ru/Cur nanodots was evaluated in IR-induced AKI and the progression from IR-AKI to CKD.

## 2 Materials and methods

### 2.1 Materials

Ruthenium (III) chloride hydrate and curcumin were purchased from Energy Chemical. Polyvinylpyrrolidone (PVP) was purchased from Sigma-Aldrich. MitoSOX™ Mitochondrial Superoxide Indicator (396/610 nm) was purchased from Invitrogen. The Bicinchoninic acid (BCA) protein assay kit, CCK-8, and DAPI were purchased from Beyotime Biotechnology. Anti-KIM-1 (PA5-98302) antibody was purchased from Invitrogen. Anti-nitrotyrosine (ab61392) was purchased from Abcam. Anti-NGAL (30700-1-AP), anti-F4/80 (28463-1-AP), anti-MPO (22225-1-AP), anti-PPARγ (16643-1-AP), anti-α-SMA (14395-1-AP), anti-TGF-β1 (21898-1-AP) antibodies were purchased from Proteintech. Superoxide dismutase (SOD) and malondialdehyde (MDA) kits were purchased from Nanjing Jiancheng Bioengineering Institute. TNF-α, IL-6, IL-1β and TGF-β1 enzyme-linked immunosorbent assay (ELISA) kits were purchased from BOSTER Biological Technology.

HK-2 cells (RRID: CVCL 0302) were purchased from Pricella Life Science and Technology Co., Ltd., and cultured in DMEM/F12 medium supplemented with 10% fetal bovine serum and 1% penicillin/streptomycin, maintained at 37°C in a humidified atmosphere with 5% CO_2_.

### 2.2 Synthesis and characterization of Ru/Cur nanodots

**FIGURE 2:**
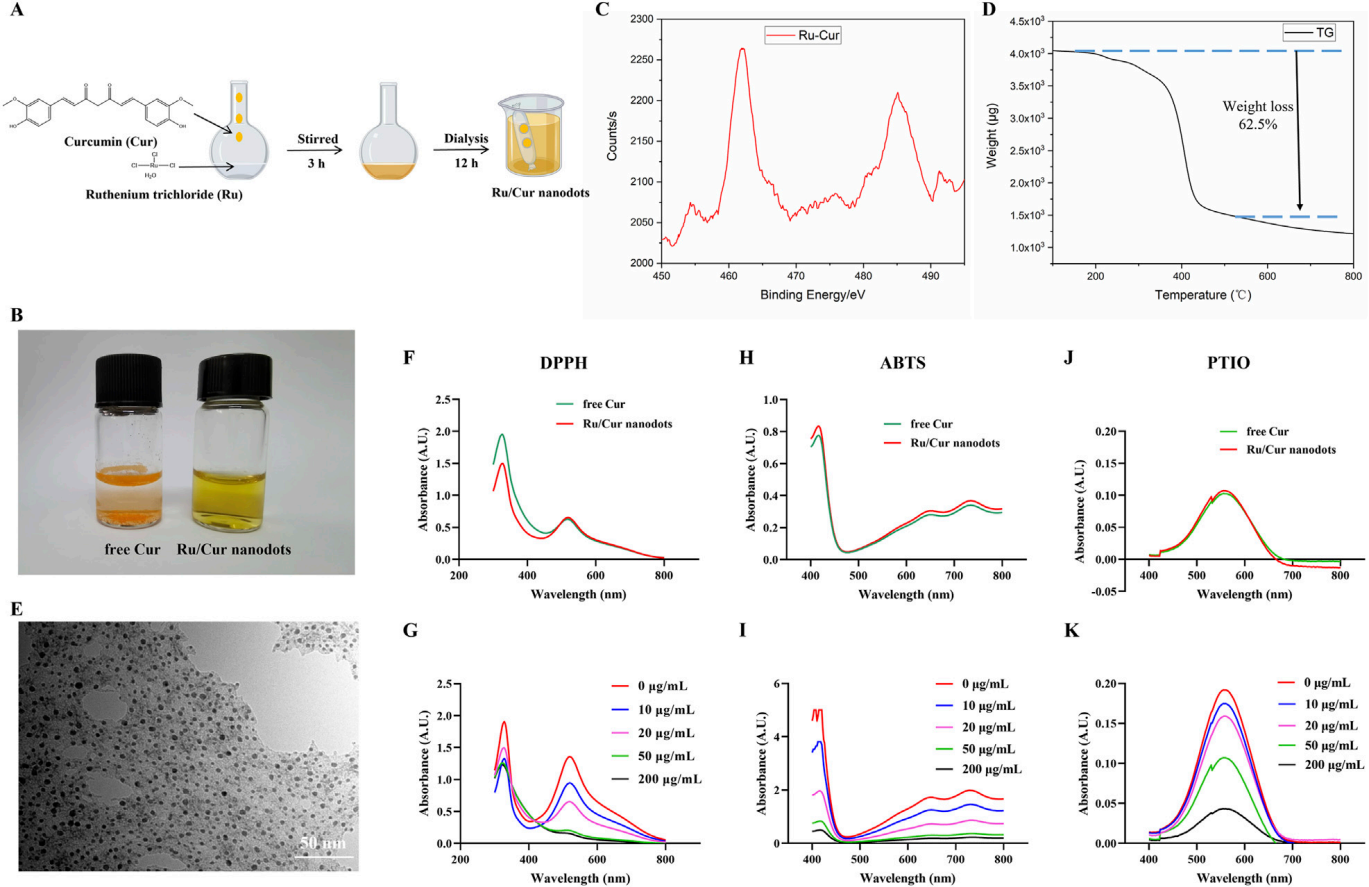
Characterization of Ru/Cur nanodots. **(A)** Synthetic procedure of Ru/Cur nanodots. **(B)** Optical images of free Cur and Ru/Cur nanodots. **(C)** XPS spectra of Ru in Ru/Cur nanodots. **(D)** TG curve of Ru/Cur nanodots. **(E)** TEM image of Ru/Cur nanodots. **(F)** Representative absorption spectra of free Cur and Ru/Cur nanodots scavenging DPPH. **(G)** Representative absorption spectra show that Ru/Cur nanodots dose-dependently scavenge DPPH. **(H)** Representative absorption spectra show the scavenging of ABTS by free Cur and Ru/Cur nanodots. **(I)** Representative absorption spectra show that Ru/Cur nanodots dose-dependently scavenge ABTS. **(J)** Representative absorption spectra show the scavenging of PTIO by free Cur and Ru/Cur nanodots. **(K)** Representative absorption spectra of Ru/Cur nanodots dose-dependently scavenging PTIO.

The Ru/Cur nanodots were synthesized by mixing ruthenium salt with curcumin and stirring at room temperature (Figure 2A). In short, 20 mg of ruthenium trichloride hydrate was dissolved in 1 mL of methanol, and then a 5 mL methanol solution containing 66 mg of polyvinylpyrrolidone (PVP) was added drop by drop. Then a 1 mL methanol solution containing 10 mg of Cur was added dropwise and stirred for another 3 h. The Ru/Cur nanodots were obtained by dialysis in water overnight and stored at 4°C for use.

Fourier transform infrared (FT-IR) spectra were measured on a Thermo NICOLET6700 spectrophotometer using KBr. A 1–2 mg sample of Cur or Ru/Cur nanodots was mixed with an appropriate amount of potassium bromide, respectively. Place the mixture into a mold and press it into a transparent sheet using a hydraulic and oil press. The UV-visible absorbance was measured on a Shimadzu UV-2600 spectrometer. The samples of Cur and Ru/Cur complexes for UV absorption were diluted to a concentration of 5.0 × 10^−5^ mol/L in anhydrous methanol. Particle size and distribution were measured by dynamic light scattering (DLS). Transmission electron microscopy (TEM) images were captured using a Hitachi HT-7800 TEM. Thermogravimetric analysis was conducted with a Hitachi 7200. The samples of Cur and Ru/Cur complex were heated from 25°C to 900°C at a rate of 10°C/min under a nitrogen gas flow of 30 mL/min.

### 2.3 Free radical scavenging test

#### 2.3.1 DPPH

Incubate 400 μL of DPPH solution (0.5 mg/mL) in 4 mL of ethanol, containing Ru/Cur nanodots at different concentrations (0, 10, 20, 50, 200 μg/mL), with three replicates for each concentration. Place the samples in a light-protected environment at room temperature for 2 h. Subsequently, the full spectrum scan from 300 to 800 nm was measured for one set of samples using a UV-Vis spectrophotometer (Neisi et al., 2024).

#### 2.3.2 ABTS

Mix equal volumes of ABTS diammonium salt (7.4 mM) and potassium persulfate solution (2.6 mM) at room temperature, protected from light, and let the mixture stand for 12 h. Then, dilute the mixture 20 times with deionized water to reduce its absorbance to below 1.0 at 734 nm, obtaining the ABTS radical working solution. Take 400 μL of the ABTS radical working solution and add different concentrations of Ru/Cur nanodots (0, 10, 20, 50, 200 μg/mL). Each concentration should have three parallel groups. Add 400 μL of PBS and finally top up with deionized water to a total volume of 4 mL.

#### 2.3.3 PTIO

Incubate 400 μL of freshly prepared PTIO (0.5 mg/mL) solution in 4 mL of PBS, containing different concentrations of Ru/Cur nanodots (0, 10, 20, 50, 200 μg/mL). Each concentration should have three parallel groups. Mix thoroughly and incubate at room temperature, protected from light, for 24 h. Then, the full absorption spectrum from 300 to 800 nm was measured using UV-visible spectroscopy (He et al., 2023).

### 2.4 Cellular uptake

After cells were incubated with 200 µM H2O2 for 2 h, Ru/Cur nanodots solution was added to achieve a final concentration of 2 μg/mL. At 1, 2, and 6 h post-incubation, the medium was discarded, and the cells were washed thrice with ice-cold PBS. Subsequently, cells were fixed with 4% paraformaldehyde solution for 15 min, and stained with DAPI for 5 min. The internalization of the compounds was observed using inverted fluorescence microscope (Axio Observer 5, ZEISS).

### 2.5 CCK-8

The viability of HK-2 cells was assessed using a CCK-8 assay. HK-2 cells were seeded in 96-well cell culture plates at a density of 5×10^3^ cells per well, and cultured at 37°C in an atmosphere of 5% CO_2_ for 24 h. Cells were incubated with 200 µM H_2_O_2_ for 2 h, and then treated with Ru/Cur nanodots for 24 h, Cur as control. Following the addition of the CCK-8 reagent, the plate was incubated at 37°C for 2 h. Absorbance at 450 nm was then measured using a microplate reader (Multiskan FC, Thermo).

### 2.6 MitoSOX red

To explore the intracellular ROS production, HK-2 cells were treated with Ru/Cur nanodots and examined using MitoSox Red indicator. HK-2 cells were seeded in 24-well cell culture plates at a density of 2 × 104 cells per well and cultured at 37°C in an atmosphere of 5% CO2 for 24 h. Cells were then incubated with 200 µM H2O2 for 2 h. Following this, they were treated with Ru/Cur nanodots for 24 h, with Cur used as a control. The cells were washed three times with PBS and incubated with MitoSox Red (10 μM) for 30 min. MitoSox Red fluorescence signals (Ex/Em, 396/610 nm) were observed using an inverted fluorescence microscope. MitoSox Red fluorescence signals were also detected by the flow cytometer.

### 2.7 Animals

The Balb/c mice (6 weeks, 20–25 g) were obtained from SpePharm (Beijing) Biotechnology Co., Ltd. The experimental process was performed under the approval of the Animal and Medical Ethics Committee of Zhejiang Huitong Test & Evaluation Technology Group Co., Ltd., and the ethics approval number was HT-2024-LWFB-0018.

### 2.8 Experimental procedure for IR-AKI or IR-AKI to CKD mice

To create the IR-AKI mice, procedures from previous studies were followed (Huang et al., 2024). The mice’s body temperature was consistently maintained at 37°C throughout the process. A midline abdominal incision was made to clamp both renal pedicles for 40 min. The successful occlusion of blood flow was verified by observing a change in renal color. A significant increase in CRE and BUN levels was observed at 24 h after IR, establishing the reperfusion duration at 24 h. Ru/Cur nanodots or free Cur (Cur, 5 mg/kg) were administered to IR-AKI mice 100 min before reperfusion.

To explore the renoprotective effects of Ru/Cur nanodots on the progression from AKI to CKD, we began by identifying the onset of renal fibrosis following IR-AKI. Fourteen days post-IR, when CRE and BUN levels had nearly returned to baseline, Masson’s trichrome staining was used to detect collagen, the sign of interstitial fibrosis in kidney tissues. Consequently, we set the evaluation period for renal fibrosis at 14 days post-IR. Ru/Cur nanodots were administered intravenously at a dose of 5 mg/kg Cur to IR-AKI to CKD mice, three times every other day, starting from the first day post-IR.

### 2.9 Biodistribution

To explore the distribution of Ru/Cur nanodots, mice were administered an intravenous injection of Ru/Cur nanodots or free Cur at a dosage of 5 mg/kg Cur. At 1, 6 and 24 h post-administration, the mice were euthanized, and their organs were harvested, sectioned, embedded, and sealed for analysis.

### 2.10 Kidney function

Mice in each treatment group were euthanized 24 h after reperfusion, and body weight was monitored in each group. Blood was collected via orbital puncture, and the serum was separated by centrifugation at 2000 g for 15 min. Serum levels of CRE and BUN were analyzed using an automatic biochemical analyzer.

### 2.11 Histopathological examination

Kidney tissues were washed with PBS, fixed, dehydrated, embedded, sectioned, and stained with hematoxylin and eosin (H&E). The dehydration process involved sequential treatment with ultrapure water and ethanol. After embedding, sections were smoothed in 42°C water and then stained with H&E, which included hematoxylin staining, differentiation, bluing, eosin staining, and other steps (Usefzay et al., 2022). Finally, slides were mounted with neutral gum and observed and photographed.

### 2.12 Oxidative stress

SOD and MDA are crucial markers of oxidative stress, indicating the kidney’s free radical scavenging capability and lipid peroxidation level, respectively. The activity of SOD and the levels of MDA in kidney tissue homogenates were assessed using commercial kits following the provided instructions (Fu et al., 2024).

### 2.13 Proinflammatory cytokines

To examine renal proinflammatory cytokine levels, TNF-α and IL-6 concentrations in kidney tissue homogenates were measured employing ELISA kits, following the procedures outlined by the manufacturer (Alsabaani et al., 2024).

### 2.14 Immunofluorescent staining

After incubating cells or kidney frozen sections (8 μm) with blocking buffer (containing 5% normal goat serum and 0.3% Triton-100 in PBS) for 1 h, the blocking buffer was removed, and the sections were incubated with primary antibodies overnight at 4°C in a humid chamber. Following PBS washes to remove unbound primary antibodies, secondary antibodies labeled with DAPI were added and incubated at room temperature in the dark for 2 h. After washing, the sections were mounted and sealed. Finally, fluorescence signals were captured using an inverted fluorescence microscope, and fluorescence signals analyzed semi-quantitatively (Luo et al., 2021).

### 2.15 Safety

After administering Ru/Cur nanodots via intravenous injection, mice were euthanized at designated time points (day 1, 7 and 28), and their major organs (liver, spleen, kidney, heart, and lung) were harvested for histological examination. Tissue sections were stained with H&E and inspected under a light microscope for imaging analysis. Comprehensive hematological and biochemical assays were performed on whole blood and serum samples to evaluate physiological and biochemical parameters.

### 2.16 Statistics

Experimental data are presented as means ± standard deviation (SD). Comparisons across multiple groups were conducted using one-way analysis of variance, while pairwise comparisons between groups were performed using the *t*-test. P < 0.05 was considered to indicate statistical significance.

## 3 Results and discussion

### 3.1 Characterization of Ru/Cur nanodots

Dissolve Cur in methanol, then add it dropwise to a methanol solution containing iron ions and PVP while stirring. After overnight dialysis in water to remove unreacted ions, the solution transitions to the aqueous phase. Compared to free Cur, Ru/Cur nanodots exhibit excellent water solubility (Figure 2B). The Ru component in Ru/Cur nanodots was analyzed using X-ray photoelectron spectroscopy (XPS). As shown in Figure 2C, the two strong binding peaks at 462 eV and 485 eV indicate the presence of trivalent Ru 3p3/2 and 3p1/2 in Ru/Cur nanodots. Thermal gravimetric analysis (TGA) showed a molar ratio of Ru to Cur in Ru/Cur nanodots to be 1:2, implying that two Cur molecules coordinate with one ruthenium ion, as shown in Figure 2D.To further elucidate the coordination relationship between the ruthenium ion and curcumin molecules, FT-IR was performed on the dried powders of Cur molecules and Ru/Cur nanodots as shown in Supplementary Figure S1A significant decrease in the stretching vibration peak of HO-C in the range of 1,150–1,200 cm-1 suggests that the hydroxyl groups of Cur molecules are bound to ruthenium through coordination.

Through TEM observation and dynamic light scattering detection (Figure 2E and Supplementary Figure S2), Ru/Cur nanodots with an approximate particle size of 5.5 ± 1.3 nm were seen to be uniformly distributed across the visual field. Without any surface modifications, the Ru/Cur nanodots exhibited excellent water solubility and remarkable stability in diverse physiological environments, including water, PBS, and DMEM/F12 culture medium containing 10% fetal bovine serum, with a hydrated particle size consistently around 5 nm (Supplementary Figure S3).

The antioxidative abilities of Ru/Cur nanodots were evaluated using DPPH as an antioxidant indicator. The absorption peak of DPPH at 517 nm, a characteristic feature, diminished or disappeared in the presence of free radical scavengers. When the same concentration of Ru/Cur nanodots and free Cur were added to the DPPH solution, there was a similar decrease in DPPH absorption at 517 nm. This outcome suggested that metal-organic coordination did not impair the antioxidant activity of Cur (Figure 2F). Furthermore, Ru/Cur nanodots demonstrated dose-dependent radical scavenging activity (Figure 2G). Additionally, Ru/Cur nanodots were capable of scavenging ABTS (Figures 2H, I) and PTIO (Figures 2J, K) in a similar manner.

**FIGURE 3:**
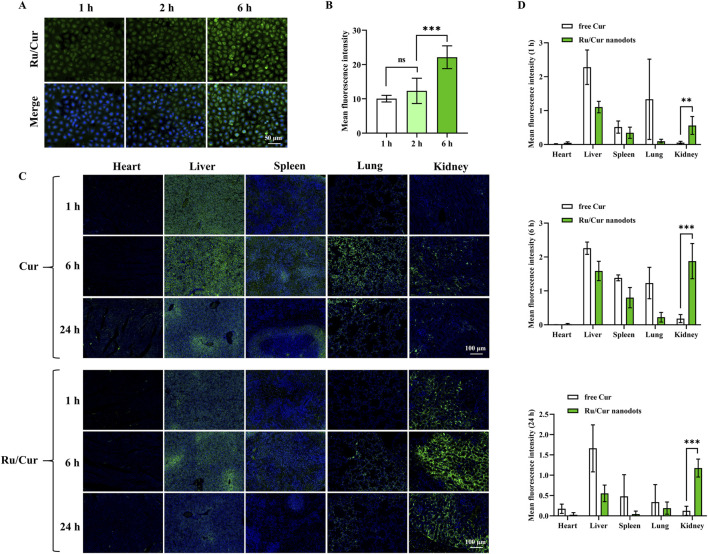
Tissue biodistribution of Ru/Cur nanodots *in vitro* and *in vivo*. **(A)** Representative fluorescence images of cellular uptake of Ru/Cur nanodots in H2O2-induced HK-2 cells after incubation for 1 h, 2 h and 6 h. **(B)** The mean fluorescence intensity of **(A)**. **(C)** Representative fluorescence images of free Cur and Ru/Cur nanodots in IR-AKI mice at 1 h, 6 h and 24 h post-injection. **(D)** The mean fluorescence intensity at **(C)** time points (1 h, 6 h, and 24 h) was found to be statistically significant with *P < 0.05, **P < 0.01, and ***P < 0.001.

The fluorescence signal demonstrated a time-dependent internalization of Ru/Cur nanodots within the cells (Figures 3A, B), showing that Ru/Cur nanodots could be effectively internalized by HK-2 cells. Additionally, various concentrations of Ru/Cur nanodots were added to HK-2 cells and incubated for 48 h to evaluate their impact on cell viability. Results from the CCK-8 assay revealed that the cell viability remained above 80% even at Ru/Cur nanodots concentrations of up to 100 μg/mL (Supplementary Figure S4). Furthermore, optical microscopy observations of cell morphology revealed no significant changes (Supplementary Figure S5). These findings clearly show that Ru/Cur nanodots have low levels of cytotoxicity.

### 3.2 *In vivo* biodistribution of Ru/Cur nanodots

In the *in vivo* experiments, the biodistribution of Ru/Cur nanodots was analyzed. Ru/Cur nanodots were administered via tail vein injection, with free Cur used as a control to investigate the distribution of the drug at 1, 6 and 24 h post-injection. As shown in Figures 3C, D, the results indicated that Ru/Cur nanodots were present in the renal tissues of IR-AKI mice at 1 h post-injection, suggesting that Ru/Cur nanodots exhibited rapid and efficient distribution in the kidneys. By contrast, little fluorescence signal of free Cur was observed in kidney tissues. The renal distribution of Ru/Cur nanodots increased further at 6 h post-injection. However, by 24 h post-injection, the fluorescence signals in kidney tissues of IR-AKI mice had decreased compared to the levels observed at 6 h. Additionally, compared with free Cur, less distribution of Ru/Cur nanodots was observed in other tissues, including the heart, liver, spleen, and lungs. These findings clearly demonstrated the superior targeting capability of Ru/Cur nanodots towards renal tissues, highlighting its potential for the targeted treatment of IR-AKI.

### 3.3 Ru/Cur nanodots mitigates tubular cell damage

**FIGURE 4:**
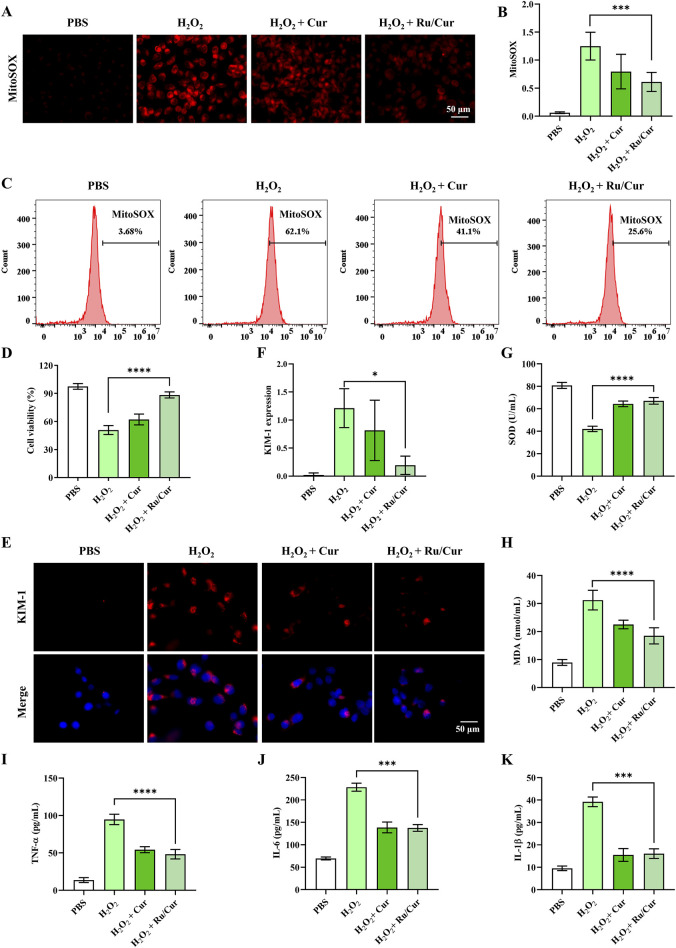
Ru/Cur nanodots protected HK-2 cells from oxidative damages. **(A)** The changes in mitochondrial ROS production in H_2_O_2_-induced cells after treatments. **(B)** The mean fluorescence intensity of **(A)**. **(C)** The changes in mitochondrial ROS production detected by flow cytometer. **(D)** The changes in cell viability of HK-2 cells. **(E)** Immunofluorescence staining on the expression of KIM-1 in HK-2 cells. **(F)** The mean fluorescence intensity of **(F)**. **(G,H)** The changes of SOD and MDA concentrations in HK-2 cells. **(I,J)** and **(K)** The changes of TNF-α, IL-6 and IL-1β concentrations in HK-2 cells. Scale bar, 50 μm. Data are presented as means ± SD.*P < 0.05, ***P < 0.001, and ****P < 0.0001.

Based on the aforementioned findings, it was evident that Ru/Cur nanodots possessed a potent capacity to mitigate ROS and enhance cell viability. MitoSOX Red is a live-cell fluorescent probe specifically targeting mitochondria, with the ability to penetrate cell membranes. Once inside the mitochondria, MitoSOX Red is oxidized by superoxide, unaffected by other ROS or RNS generating systems. The oxidized MitoSOX Red then binds to nucleic acids within the mitochondria or the cell nucleus, emitting strong red fluorescence. Therefore, MitoSOX Red can be used as a fluorescent indicator specifically designed for detecting superoxide. Fluorescence imaging (Figures 4A, B) revealed that Ru/Cur nanodots significantly reduced intracellular ROS levels in HK-2 cells subjected to H_2_O_2_-induced oxidative stress, compared to cells treated with H_2_O_2_ alone. The fluorescence signals of MitoSOX Red were also detected by flow cytometer, and the results (Figure 4C) showed that Ru/Cur nanodots effectively reduced intracellular ROS levels.

Additionally, the CCK-8 assay demonstrated that Ru/Cur nanodots markedly improved cell survival rates following H2O2 exposure (Figure 4D). These results collectively indicate that Ru/Cur nanodots effectively scavenge ROS, thereby providing a protective effect against oxidative damage and promoting cell survival. This highlights the potential therapeutic application of Ru/Cur nanodots in conditions characterized by elevated ROS, such as IR-AKI. We subsequently investigated whether Ru/Cur nanodots alleviated H2O2-induced cytotoxicity in renal tubules. After being incubated with H2O2, the expression of kidney injury molecule-1 (KIM-1), a known marker of tubular cytotoxicity, increased. However, this elevation was significantly suppressed after treatment with Cur or Ru/Cur nanodots, as shown by the results of immunofluorescence (Figures 4E, F).Ru/Cur nanodots effectively ameliorated oxidative stress by reducing levels of SOD and MDA (Figures 4G, H), and also decreased proinflammatory factors such as TNF-α, IL-6, and IL-1β (Figures 4I–K).

### 3.4 Ru/Cur nanodots ameliorate renal pathology and oxidative stress in IR-AKI mice

**FIGURE 5:**
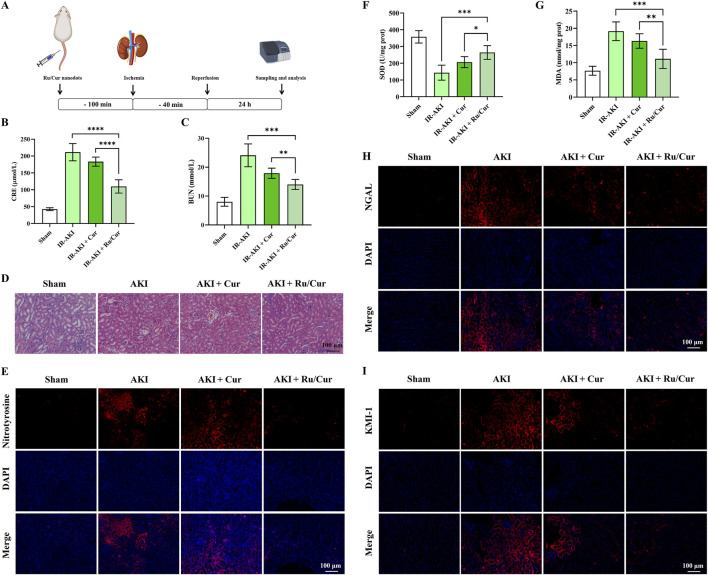
Ru/Cur nanodots ameliorate renal pathology and oxidative stress in IR-AKI mice. **(A)** A schematic summary of the administration schedule used for Cur or Ru/Cur nanodots against IR-AKI. **(B, C)** The changes of CRE and BUN in IR-AKI mice (n = 6). **(D)** H&E staining of kidney tissues revealed the overall structure and morphology of the tissue. **(E)** Immunofluorescence staining was performed to assess the expression of nitrotyrosine in kidney tissues, providing insight into oxidative stress levels in the tissue. **(F)** The changes of SOD and MDA concentrations in kidney tissues were measured in a sample size of 6. **(G)** Additionally, immunofluorescence staining was performed to assess the expression of KIM-1 and NGAL in kidney tissues. Scale bar, 100 μm. Data are presented as means ± SD.*P < 0.05, **P < 0.01, ***P < 0.001, and ****P < 0.0001. **(H,I)** Immunofluorescence staining on the expression of KIM-1 and NGAL in kidney tissues.

As shown in Figure 5A, the schedule of Ru/Cur nanodots or Cur administration was set at 100 min before reperfusion, and the sampling and analysis were performed at 24 h post-reperfusion. Ru/Cur nanodots effectively restored kidney function in IR-AKI mice, whereas the same dose of Cur had significantly less therapeutic effect, including CRE (Figure 5B) and BUN (Figure 5C).H&E staining of kidney tissues in IR-AKI mice revealed severe tubular damage, including significant tubular dilation and deformation, presence of numerous cellular debris in the lumen, particularly loss of the brush border in proximal tubular epithelial cells, swelling, necrosis or detachment of epithelial cells, and some cells showing deep staining and shrinkage of nuclei. Compared with Cur, treatment with Ru/Cur nanodots effectively alleviated these tubular injuries (Figure 5D).

The increased ROS levels during IR injury promoted the formation of nitrotyrosine. Therefore, the accumulation of nitrotyrosine can serve as a marker of oxidative stress (Shinbo et al., 2013). The accumulation of nitrotyrosine may be associated with inflammatory responses, activating various inflammatory signaling pathways, promoting the recruitment of inflammatory cells, and releasing inflammatory factors, all of which further exacerbate kidney injury. Thus, nitrotyrosine is not only a marker of oxidative stress but may also play an important role in the inflammatory response during IR-AKI. As shown in Figure 5E, the positive expression of nitrotyrosine was observed in kidney tissue of IR-AKI mice. Ru/Cur nanodots effectively reduced the expression of nitrotyrosine, whereas the same dose of Cur had significantly less therapeutic impact. Further validation of the ROS scavenging efficacy of Ru/Cur nanodots was done through measures of SOD (Figure 5F) and MDA (Figure 5G) activity, and the expression of KIM-1 (Figure 5H) and neutrophil gelatinase-associated lipocalin (NGAL) (Figure 5I) in kidney tissues. ROS exacerbate tubular damage by reducing SOD enzyme activity and oxidizing lipids in kidney tissues, leading to elevated expression of KIM-1 and NGAL. Ru/Cur nanodots showed efficacy in reversing these indicators compared to the IR-AKI and Cur group, further confirming their therapeutic effects in IR-AKI mice.

### 3.5 Ru/Cur nanodots suppresses inflammation in IR-AKI mice

**FIGURE 6:**
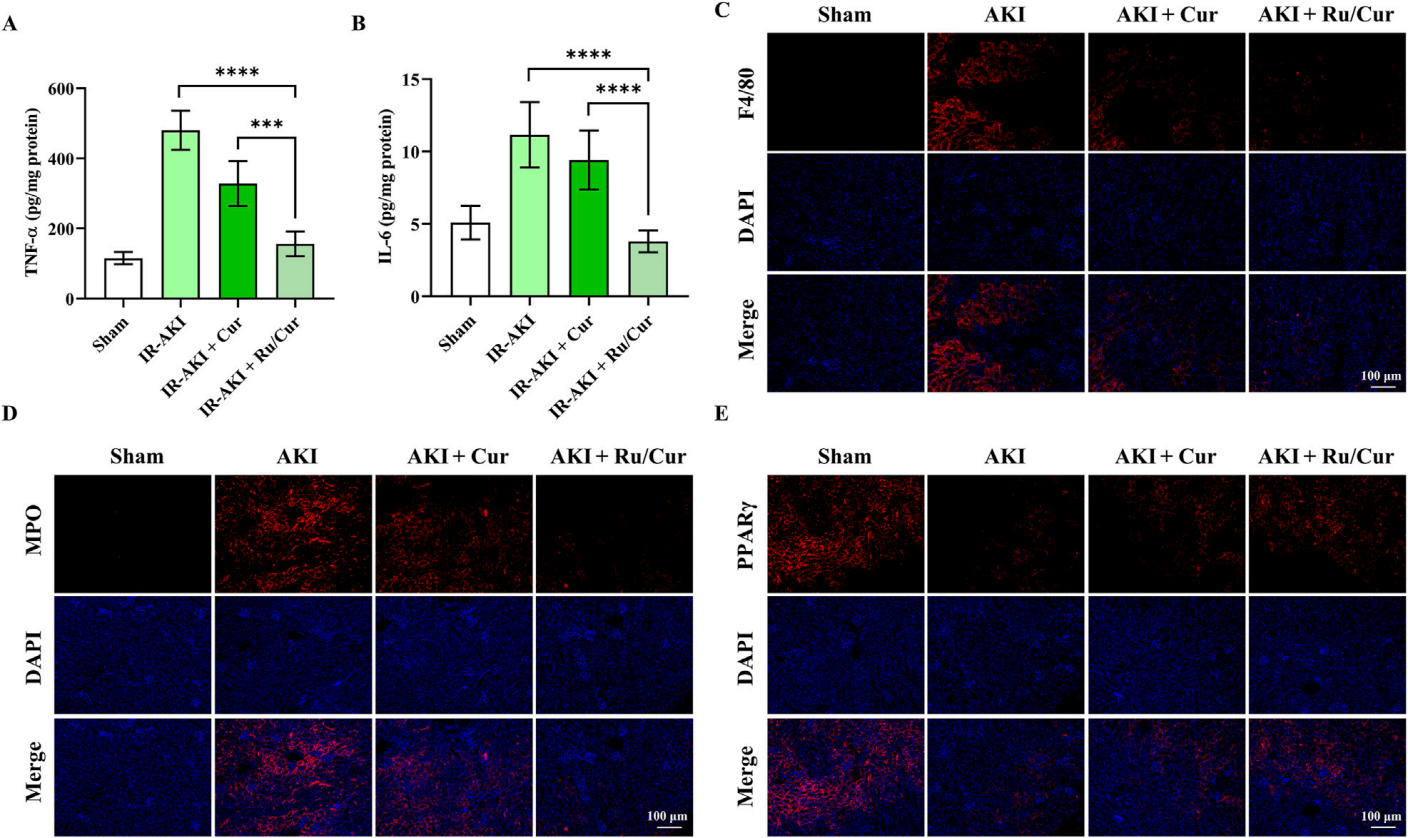
Ru/Cur nanodots suppress inflammation in IR-AKI mice. **(A,B)** The changes of TNF-ɑ and IL-6 concentrations in kidney tissues (n = 6) were analyzed. **(C–E)** Immunofluorescence staining on the expression of F4/80, MPO and PPARγ in kidney tissues. Scale bar, 100 μm. Data are presented as means ± SD.***P < 0.001, and ****P < 0.0001.

In this study, we also explored the anti-inflammatory properties of Ru/Cur nanodots. Following IR-AKI, there was a notable reduction in the expression of inflammatory cytokines, including TNF-α (Figure 6A) and IL-6 (Figure 6B), in mice treated with Ru/Cur nanodots. To evaluate the infiltration of inflammatory cells in renal tissues, we conducted immunostaining for F4/80 (Figure 6C) and MPO (Figure 6D), markers for macrophages and neutrophils, respectively. It was found that Ru/Cur nanodots effectively mitigated the IR-induced cell infiltration. Additionally, there was a significant upregulation of peroxisome proliferator-activated receptor γ (PPARγ) in the IR-AKI mice treated with Ru/Cur nanodots (Figure 6E). This increase in PPARγ expression was associated with Cur’s anti-inflammatory activity.

### 3.6 Ru/Cur nanodots prevent IR-AKI to CKD

**FIGURE 7:**
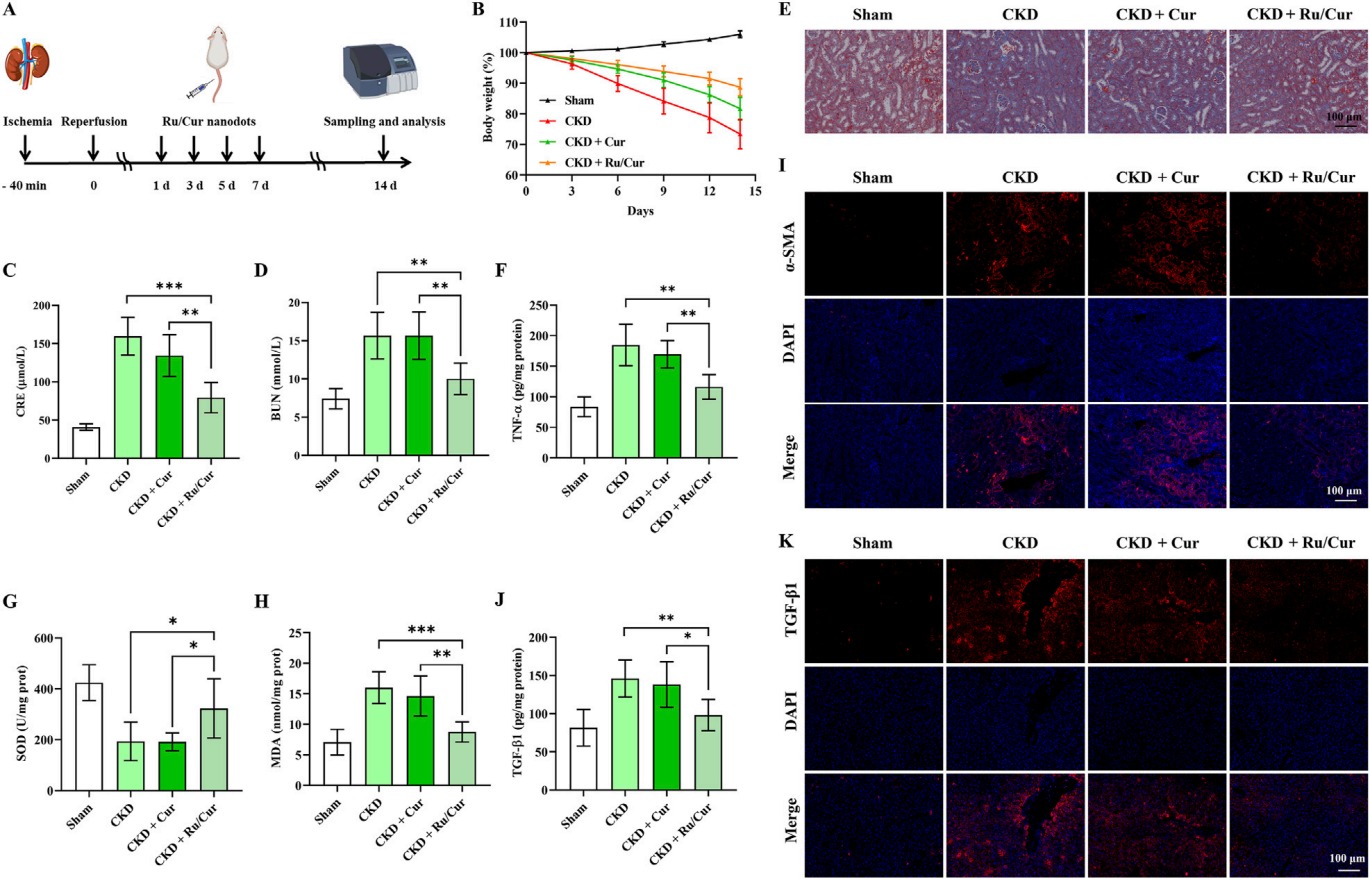
Ru/Cur nanodots prevent IR-AKI to CKD. **(A)** A schematic summary of the administration schedule used for Cur or Ru/Cur nanodots against IR-AKI to CKD is presented. **(B)** The changes of body weight (n = 6) were also monitored throughout the study. **(C)** The changes of CRE and BUN in CKD mice (n = 6) were analyzed. **(D)** The results showed a significant increase in both CRE and BUN levels. **(E)** Masson’s trichrome staining of kidney tissues was also performed to assess histological changes. **(F)** The changes in TNF-ɑ concentrations in kidney tissues (n = 6). **(G)** and **(H)** The changes in SOD and MDA concentrations in kidney tissues (n = 6). **(I)** Immunofluorescence staining on the expression of α-SMA in kidney tissues. **(J)** The changes of the TGF-β1 concentrations in kidney tissues (n = 6). Immunofluorescence staining was performed to analyze the expression of TGF-β1 in kidney tissues. The scale bar used for the images is 100 μm.Data are presented as means ± SD. *P < 0.05, **P < 0.01, and ***P < 0.001. **(K)** Immunofluorescence staining on the expression of TGF-β1 in kidney tissues.

Following this treatment schedule (Figure 7A), we administered Ru/Cur nanodots (Cur, 5 mg/kg) to mice transitioning from IR-induced AKI to CKD. CKD gradually destroys normal kidney tissue and leads to loss of renal function. During the progression of renal fibrosis, weight gain is a common phenomenon, attributable to several factors including metabolic changes, reduced appetite, protein loss, malnutrition, chronic inflammation with increased metabolic consumption, and fluid and electrolyte imbalances. As shown in Figure 7B, Ru/Cur nanodots effectively improved weight gain in CKD mice. The changes in CRE and BUN levels were detected in CKD mice after being treated with Ru/Cur nanodots. The Ru/Cur nanodots demonstrated superior efficacy in improving the levels of CRE (Figure 7C) and BUN (Figure 7D) compared to Cur alone, suggesting that they possess an effective protective function on renal function. Histopathological examinations using Masson’s trichrome staining (Figure 7E) revealed lower collagen accumulation in the group treated with Ru/Cur nanodots compared to the AKI to CKD model. TNF-α is a major mediator of chronic inflammatory responses. CKD is often accompanied by low-grade chronic inflammation in the kidneys and throughout the body, and TNF-α plays a crucial role in this inflammatory response. TNF-α activates inflammatory signaling pathways such as nuclear factor-kappa B (NF-κB), promoting the release of inflammatory factors, exacerbating kidney damage, and advancing the process of fibrosis. As shown in Figure 7F, TNF-ɑ concentrations in kidney tissues were significantly reduced in CKD mice treated with Ru/Cur nanodots. The antioxidative capacity of the kidneys is typically impaired during CKD, leading to a reduction in SOD activity. The exacerbation of oxidative stress results in an increased peroxidation of renal cell membrane lipids, thereby elevating MDA levels. It was observed that Ru/Cur nanodots could significantly enhance SOD activity (Figure 7G) and reduce MDA levels (Figure 7H), thereby mitigating renal damage caused by oxidative stress. Compared to the use of Cur alone, Ru/Cur nanodots exhibited a more potent antioxidative effect, which further substantiated their potential in the treatment of CKD.

During the CKD progression, interstitial cells in the kidney can differentiate into myofibroblasts. These myofibroblasts express α-smooth muscle actin (α-SMA) and have the ability to synthesize and secrete extracellular matrix components such as collagen. Therefore, α-SMA is often used as a marker of renal fibrosis. Immunofluorescence staining for ɑ-SMA was performed to investigate whether Ru/Cur nanodots had antifibrotic effects. The results (Figure 7I) showed that the expression of ɑ-SMA in kidney tissues was effectively reduced in CKD mice treated with Ru/Cur nanodots. The expression of TGF-β1 was also detected to investigate if Ru/Cur nanodots had antifibrotic effects by suppressing epithelial-mesenchymal transition (EMT). Fourteen days post-IR, an increase in TGF-β1 expression was observed, which was effectively reversed in the Ru/Cur nanodots-treated group, as reflected by the results of ELISA (Figure 7J) and Immunofluorescence staining (Figure 7K).

### 3.7 The safety of Ru/Cur nanodots

**FIGURE 8:**
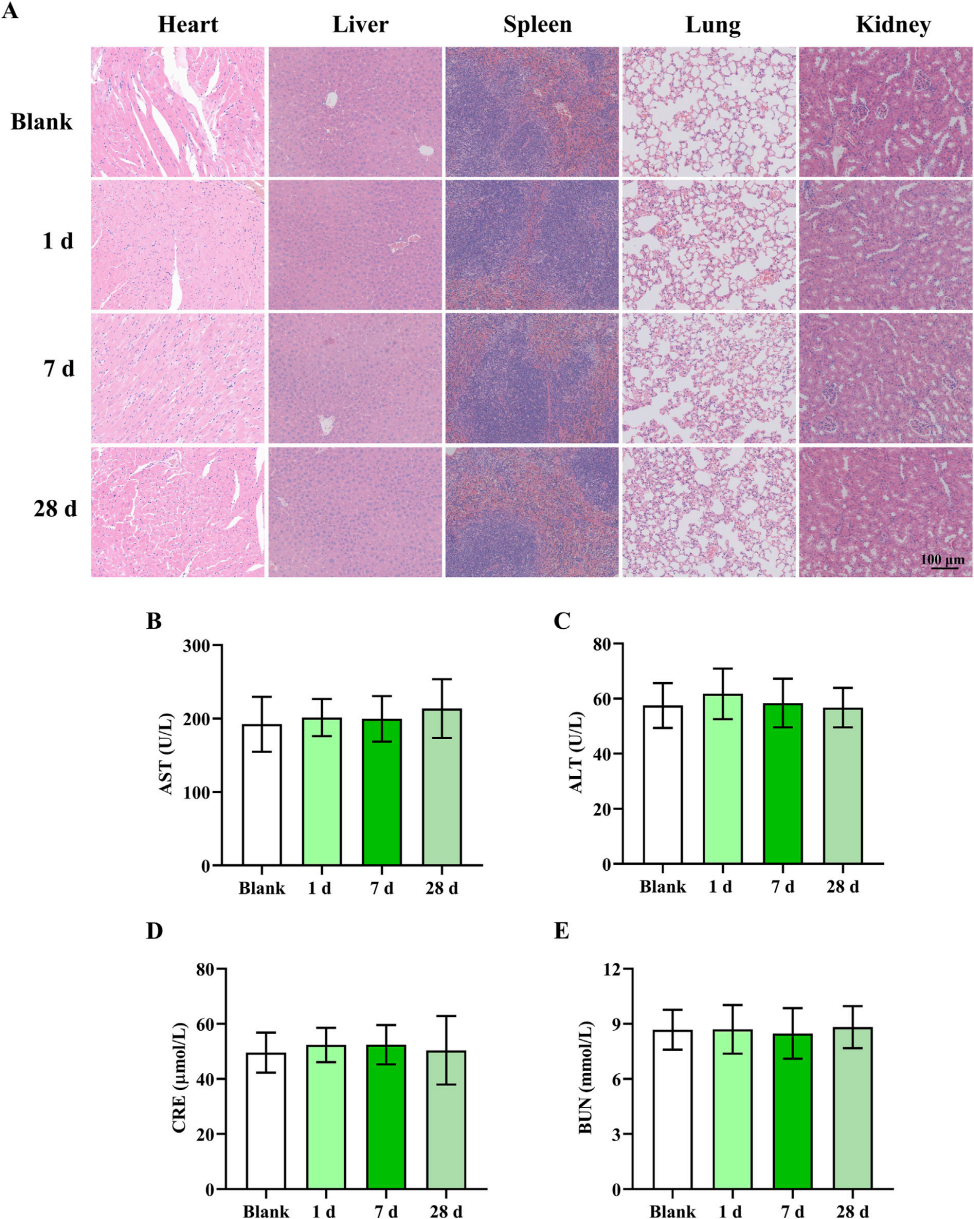
The safety of Ru/Cur nanodots. **(A)** H&E staining of heart, liver, spleen, lung and kidney. **(B,C)** The changes of AST and ALT. **(D,E)** The changes of CRE and BUN. The scale bar in the images is 100 μm. Data are presented as means ± SD.

Finally, the biosafety of Ru/Cur nanodots was evaluated in healthy mice. Mice were euthanized at different time points (1 d, 7 d and 28 d) after intravenous injection of Ru/Cur nanodots, and major organs (liver, spleen, kidney, heart, and lung) were harvested, embedded in paraffin, and stained with H&E. Observation under an optical microscope revealed no cellular tissue damage or abnormalities in the major organs of the mice (Figure 8A). The tissue structures remained intact and exhibited no significant differences compared to those of normal mice (Blank), indicating that Ru/Cur nanodots do not induce toxic effects on tissues. In addition, Ru/Cur nanodots treatment had no effects on liver function (AST, ALT) (Figures 8B, C) and kidney function (CRE, BUN) (Figures 8D, E), which were comparable to those of normal mice (Blank). This long-term safety profile is particularly important given the concerns surrounding the potential accumulation and toxicity of metal-based nanoparticles *in vivo*. Ruthenium, as a transition metal, poses certain theoretical risks of long-term bioaccumulation and associated toxicity. However, the present findings suggested that Ru/Cur nanodots, with their unique ultrasmall size and biocompatible polymer coordination structure, might mitigate these risks. The lack of observable damage or functional impairment over the course of 28 days supported the conclusion that these nanodots were well-tolerated by the body and did not induce significant toxic effects, even in long-term scenarios.

## 4 Conclusion

In this study, we demonstrate that Ru/Cur nanodots effectively eliminate various ROS and demonstrate significant therapeutic effects and biocompatibility in a mouse model of IR-AKI, reducing markers of kidney function damage, alleviating renal oxidative stress, and decreasing inflammatory cell infiltration. Ru/Cur nanodots mitigate the pathological conditions associated with both AKI and its progression to CKD by reducing IR-induced tubular cell injury. This study demonstrated that Ru/Cur nanodots possess excellent antioxidant, anti-inflammatory, and antifibrotic properties, indicating their potential clinical application value.

Based on the promising results of Ru/Cur nanodots in animal models, future studies could focus on several key developments. First, clinical translation of these nanodots could be pursued by evaluating their long-term safety and efficacy in human clinical trials for the treatment of AKI and CKD. Second, further investigation into the mechanistic insights behind the antioxidant, anti-inflammatory, and antifibrotic effects of Ru/Cur nanodots would be crucial. A deeper understanding of these mechanisms could lead to optimized formulations and enhanced therapeutic efficacy. Lastly, exploring combination therapy with other pharmacological agents, such as anti-fibrotic or anti-inflammatory drugs, may provide synergistic benefits and improve treatment outcomes for AKI and CKD. Together, these advancements would not only extend the scope of current research but also contribute to the advancement of kidney disease therapies.

## Data Availability

The original contributions presented in the study are included in the article/Supplementary Material, further inquiries can be directed to the corresponding authors.
