# Supplementary material for: Ultra-small curcumin-ruthenium coordination polymer nanodots prevent renal ischemia-reperfusion injury and the progression to chronic kidney disease
Source: Front Bioeng Biotechnol. 2025 Jan 14;12:1506909. doi: 10.3389/fbioe.2024.1506909 (PMC11772299; doi:10.3389/fbioe.2024.1506909)
Supplement: Supplementary file 1 [file DataSheet1.docx]

**Ultra-small curcumin-ruthenium coordination polymer nanodots prevent renal ischemia-reperfusion injury and the progression to chronic kidney disease**

Xian Liu ^1^ *, Qin Yu ^1^, Hai-Bo Mao ^2^, Jing-Bo Hu ^2^, Wei-Hua Liu ^1^ *

^1^ Department of Urology, Beilun People's Hospital, Zhejiang, Ningbo 315800, China.

^2^ Faculty of Materials Science and Chemical Engineering, Ningbo University, Ningbo 315211, China.

* Corresponding authors: [45103713@qq.com](mailto:45103713@qq.com), [reeseliu1995@163.com](mailto:reeseliu1995@163.com).

**Fig.1.** Fourier transform infrared spectra of Cur and Ru/Cur.


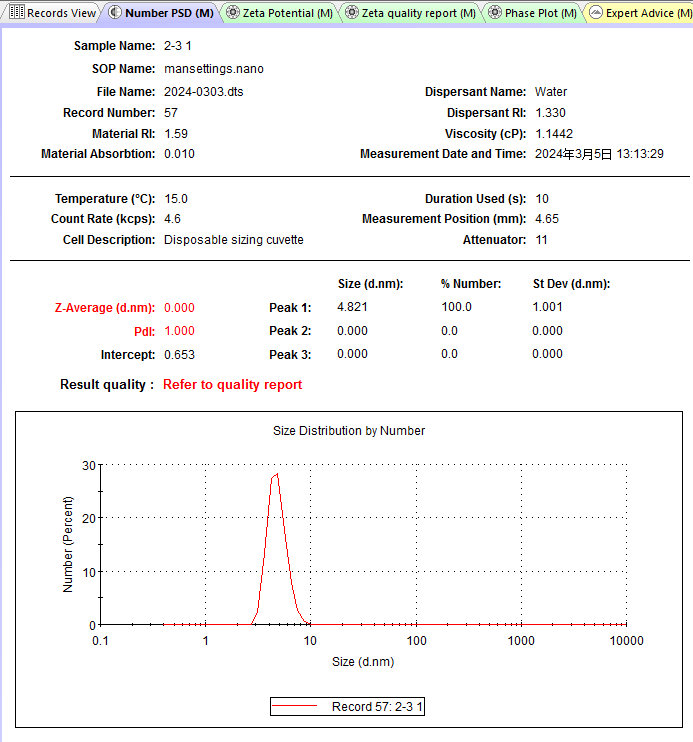


**Fig.2.** Particle size and distribution of Ru/Cur nanodots

**Fig.3.** The particle sizes of Ru/Cur nanodots in diverse physiological environments, including water, PBS, and DMEM/F12 culture medium containing 10% fetal bovine serum (n = 6), and their optical images.


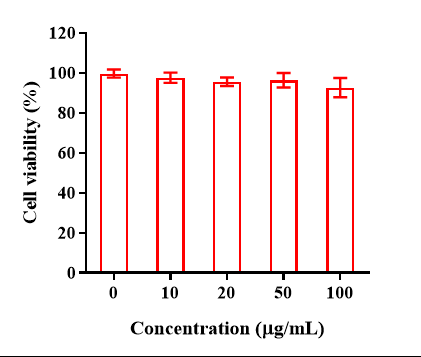


**Fig.4.** Cell viability of HK-2 cells after incubated with various concentrations of Ru/Cur nanodots for 48 h (n = 6). Data are presented as means ± SD.


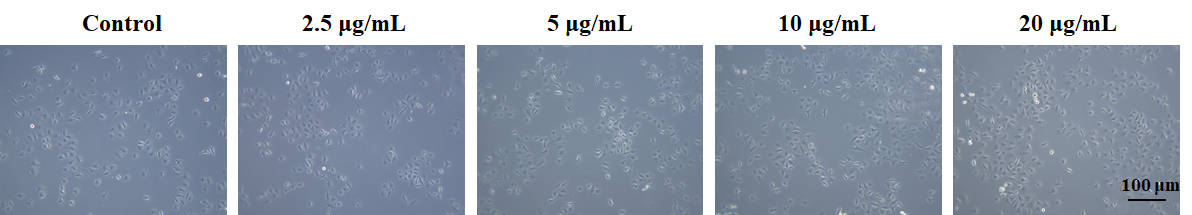


**Fig.5.** Cell morphology of HK-2 cells after incubated with various concentrations of Ru/Cur nanodots for 48 h.
